# Noninvasive NESA Microcurrent Neuromodulation for Refractory Overactive Bladder in Women: A Triple-Blind, Randomized, Sham-Controlled Pilot Trial

**DOI:** 10.3390/medicina62050936

**Published:** 2026-05-11

**Authors:** Guillermo Conde-Santos, Alicia Martín-Martínez, Sonia Carballo-Rastrilla, Abián Fernández-Mederos, Aníbal Báez-Suárez, Andrea Hernández-Pérez, María P. Quintana-Montesdeoca, Raquel Medina-Ramírez

**Affiliations:** 1 Hospital Quiron Salud Tenerife, 38006 Santa Cruz de Tenerife, Spain; gconde.urologia@gmail.com; 2 Maternal and Child Hospital of Las Palmas de Gran Canaria, 35016 Las Palmas, Spain; aliciammartinez@gmail.com (A.M.-M.); scroquet@yahoo.com (S.C.-R.); xhaplo@hotmail.com (A.F.-M.); 3 Faculty of Health Sciences, University of Las Palmas de Gran Canaria, 35016 Las Palmas, Spain; mariadelpino.quintana@ulpgc.es (M.P.Q.-M.); raquel.medina@ulpgc.es (R.M.-R.); 4 Faculty of Health Sciences, University of La Laguna, 28200 Santa Cruz de Tenerife, Spain; andrea.hernandez114@alu.ulpgc.es

**Keywords:** neuromodulation, nocturia, overactive bladder, sleep, urinary urgency

## Abstract

*Background and Objectives*: Overactive bladder (OAB) is frequently associated with impaired quality of life and sleep disturbances, particularly in women with refractory symptoms. Non-invasive neuromodulation targeting autonomic regulation has emerged as a potential therapeutic approach. The purpose of this pilot trial is to assess the efficacy and safety of NESA non-invasive neuromodulation in the treatment of patients with overactive bladder. *Materials and Methods*: Triple-blind, randomized, sham-controlled pilot trial. Women ≥ 18 years with refractory OAB were randomized to active NESA or sham using an identical protocol (10 sessions, 60 min, twice weekly). Outcomes were collected at baseline, after session 5, and after session 10. The primary outcome was mean daily micturitions, assessed using a 3-day voiding diary. Secondary outcomes included other diary variables, urinary questionnaires (ICIQ-UI SF, ICIQ-QoL, B-SAQ), and sleep measures (PSQI, ISI). *Results*: Per-protocol analysis included 43 women (NESA *n* = 24; sham *n* = 19). At session 10, mean daily micturitions decreased from 9.19 to 8.07 with NESA and increased from 10.56 to 11.03 with sham (between-group *p* = 0.043; d = −0.97). Sleep improved with NESA versus sham (PSQI *p* = 0.001; d = −0.63; ISI *p* = 0.001; d = −0.59). Between-group differences in urinary symptom questionnaires were not significant. No device-related adverse events occurred. *Conclusions*: In this pilot trial, NESA neuromodulation was safe and showed preliminary signals of benefit, particularly for mean daily frequency and sleep outcomes. However, given the exploratory design, small sample size, and per-protocol analysis, these findings should be interpreted cautiously and confirmed in larger adequately powered trials before clinical implementation.

## 1. Introduction

Overactive bladder (OAB) is a common condition in both men and women and is associated with substantial impairment in quality of life, sexual function, mental health, and sleep. According to the International Continence Society, OAB is defined as urinary urgency, usually accompanied by frequency and nocturia, with or without urinary incontinence, in the absence of urinary tract infection or other obvious pathology [1].

Population-based data indicate that OAB symptoms affect around 12% of adults, with slightly higher prevalence in women [2].

OAB is a multifactorial syndrome involving several potential pathophysiological mechanisms. Dysregulation of the autonomic nervous system has been proposed as a key contributor to lower urinary tract dysfunction, with evidence suggesting autonomic imbalance in patients with OAB [3].

In women, OAB is also frequently associated with impaired sleep quality and daytime dysfunction, even in the absence of overt nocturia, further aggravating the burden of disease [4,5].

Current non-invasive treatments for OAB include lifestyle and behavioral interventions. Pharmacological therapies such as antimuscarinics and β3-agonists are commonly used as second-line options when conservative measures are insufficient. Neuromodulation is considered when conservative strategies fail, with sacral nerve stimulation and posterior tibial nerve stimulation being the most widely used approaches [6,7,8]. These techniques, while effective, may involve invasive procedures, require repeated visits, or have limited long-term adherence, highlighting the need for alternative, minimally invasive neuromodulation strategies.

NESA neuromodulation is a non-invasive microcurrent-based system that aims to modulate autonomic nervous system activity through multiple surface electrodes placed on the limbs. The technique delivers imperceptible low-intensity, low-frequency currents in programmed sequences and has been explored in other contexts such as recovery in athletes and sleep-related disturbances [9,10,11]. Preliminary clinical observations suggested a potential improvement in lower urinary tract symptoms, which motivated the exploration of NESA neuromodulation in patients with overactive bladder syndrome.

The objective of this pilot randomized trial was to explore the efficacy and safety of non-invasive NESA neuromodulation in women with OAB. We hypothesized that NESA could improve bladder diary parameters, patient-reported symptoms, and sleep quality compared with sham stimulation.

## 2. Materials and Methods

### 2.1. Subjects

The study sample comprised 43 patients diagnosed with overactive bladder who were recruited from two hospitals in our community. Sample size estimation was based on the study by Otsuka et al. [12], using a baseline mean daily voiding frequency of 10.48 and a standard deviation of 3.06 as the reference parameters. Assuming a two-tailed comparison, 80% statistical power, and an alpha level of 0.05, the required sample size was estimated and then increased by 20% to account for potential loss to follow-up. This resulted in an estimated minimum sample of 28 participants per group after accounting for expected attrition. Given the practical constraints associated with the COVID-19 period, the study was ultimately conducted and interpreted as an exploratory pilot trial.

The study included women over 18 years of age who were diagnosed with urinary incontinence occurring three or more times per week or idiopathic overactive bladder with eight or more voids per day and/or two or more at night, while excluding other causes of bladder overactivity. Participants were required to have not obtained adequate clinical response with previous pharmacological treatments and to have performed a 4-week washout period prior to the study. Additionally, participants were required to have competent cognitive conditions. Patients were excluded from the study if they had contraindications for treatment with NESA (Neurostimulacion superficial aplicada in Spanish) neuromodulation or did not meet the inclusion criteria. The exclusion criteria encompassed pacemakers, pregnancy, internal bleeding, ulcerated skin, acute febrile processes, cancer diagnosis, phobia of electricity, and any comorbidity that could interfere with participation, adherence, or outcome assessment (e.g., severe neurological or psychiatric disorders). This trial was designed as a women-only study because recruitment occurred in urogynecology units and the target population was women with refractory idiopathic OAB; therefore, the findings are generalizable to women and may not extrapolate to men. The study period was from November 2019 to December 2021. The clinical trial design followed the CONSORT 2010 guidelines [13].

### 2.2. Study Design

This was a triple-blind, randomized sham-controlled pilot clinical trial designed to evaluate the safety and efficacy of the NESA non-invasive neuromodulation device in treating Overactive Bladder Syndrome. Participants, NESA microcurrent administrators, and the researcher who analyzed the data were blinded to the group allocation. Participants were randomly assigned to either the experimental group (active NESA) or the placebo group (sham NESA) using a fixed-size block randomization sequence generated by the data manager to ensure balanced allocation. Group assignment was concealed from participants, treating staff, and the outcome analyst. Device configuration (active vs. sham) was performed by an independent researcher not involved in treatment delivery, assessments, or statistical analysis. The study variables were collected at three different time points: before starting treatment, mid-treatment (after the 5th session), and at the end of treatment (8 weeks).

### 2.3. Procedure

The aim was to improve the mechanism of bladder function regulation, and consequently, the patients’ symptoms and quality of life. Fifty-seven women were randomized; forty-three completed the intervention and were included in the per-protocol efficacy analysis. The patients were assigned to two groups: the EG treatment (NESA microcurrents) and the PG (simulated NESA microcurrents), which were compared. Fourteen participants did not complete the protocol for various reasons (detailed in the CONSORT diagram). The per-protocol analysis included 43 women. The 24 patients in the EG completed a 10-session protocol, with each session lasting 60 min, twice a week.

The NESA microcurrent therapy is administered via wristbands and anklets, each of which is equipped with six electrodes. The total number of electrodes employed is 24, strategically positioned to cover the low-impedance areas of the limbs. In addition to this, a directional electrode is employed to direct the current flow. NESA technology is based on the application of very low-frequency microcurrents (ranging from 1.3 Hz to 14.28 Hz, depending on the program), with low intensity and voltage parameters (0.1–0.9 mA; potential difference ±3 to ±6 V). These currents are imperceptible to the patient and are intended to influence autonomic nervous system activity through distributed surface stimulation. The activation of surface-level nerves creates a flow of currents that may influence neural pathways, potentially involving central nervous system modulation, although this mechanism remains hypothetical. The coordination of these 24 electrodes, when stimulated simultaneously, produces a systemic effect rather than one that is localized to a particular muscle or nerve area. This outcome is attributed to the distributed nature of the electrode placement and the electrical parameters involved. The device was programmed according to the directions and phases outlined in this study.

**Figure 1:**
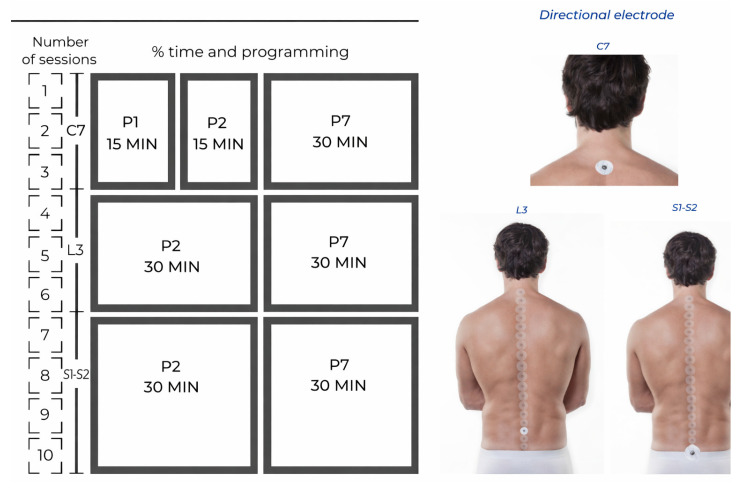
Description of the intervention protocol.

The present study was conducted in the Hospital Quironsalud and the Complejo Hospitalario Universitario Insular Materno Infantil, both located in the Canary Islands. The nursing staff from the Urogynecology service of both centers, who had no direct involvement with the participants, administered the NESA protocol in a blinded manner. Device configuration (active vs. sham) was performed by an independent researcher not involved in treatment delivery or outcome assessment. The study involved three phases. Stimulation parameters were selected based on previously published NESA protocols and clinical experience [14]. The first phase consisted of three sessions using program 1 (P1) for 15 min (from 3.85 to 7.69 Hz and an intensity within the range of 0.1 to 0.9 mA), program 2 (P2) for 15 min (from 1.14 to 1.96 Hz and an intensity within the range of 0.1 to 0.9 mA. The electrical impulses in P2 are distributed through the peripheral nerves in the ventral areas), and program 7 (P7) (from 1.92 to 14.29 Hz and an intensity within the range of 0.1 to 0.9 mA) and the directional electrode located in zone C7. The second phase was a potentiation phase that also lasted three sessions and used P2 and P7 (directional electrode placed in zone L3). The third and final phase was a maintenance phase that used P3 (from 1.14 to 1.96 Hz and an intensity within the range of 0.1 to 0.9 mA. The electrical impulses in P3 are distributed through the peripheral nerves in the dorsal areas) and P7 (directional electrode located in zone S1-S2) for the remaining sessions (Figure 1). These zones correspond to spinal segments commonly used in neuromodulation protocols (e.g., C = cervical region, L = lumbar region, S = sacral regions).

The 19 patients in the PG underwent the same NESA protocol, but their device had electrical emission switched off, making it a sham treatment. The appearance of switching on is identical to the real device. The sham intervention followed the same protocol, the same electrode placement, the same visual setup, and the same treatment duration as the active intervention, and the device appeared to be turned on in the same way. No formal assessment of blinding success was performed.

### 2.4. Outcome Measures

Bladder symptoms were assessed using a 3-day voiding diary (DM3d) validated in Spanish [15]. From the diary we obtained: total number of incontinent episodes (NIE), number of nightly voids (NNV), mean daily micturition (MDM), and mean voided volume (MVV). Participants measured voided volume using a standardized graduated container provided for diary completion.

Patient-reported outcomes included the Spanish versions of the Bladder Overactive Control Self-Assessment Questionnaire (B-SAQ) [16] and the International Consultation on Incontinence Questionnaire–Urinary Incontinence Short Form (ICIQ-UI SF) [17], with specific scores for symptoms and quality of life.

Sleep quality was measured with the Spanish version of the Pittsburgh Sleep Quality Index (PSQI) [18] and the Insomnia Severity Index (ISI) [19]. Higher scores indicate worse sleep quality and more severe insomnia.

Assessments were performed at baseline (before treatment), mid-treatment (5th session), and post-treatment (10th session).

### 2.5. Data Analysis

Data were analyzed using IBM SPSS Statistics Version 27.0 (2020). Categorical variables are presented as frequencies and percentages. Numerical variables are expressed as mean (standard deviation) and, where relevant, median and interquartile range. Normality was assessed with the Shapiro–Wilk test.

The primary analysis was conducted on a per-protocol population, including participants who completed all 10 sessions and all scheduled assessments. Given the pilot nature of the study, no formal intention-to-treat analysis was performed. This analytical approach was chosen because efficacy analyses were restricted to participants who completed the intervention and all scheduled assessments; however, it may have increased the risk of attrition-related bias.

Within-group changes over time were analyzed using nonparametric tests (Friedman test with Wilcoxon signed-rank tests for post hoc comparisons) for continuous variables. Between-group comparisons used the Mann–Whitney U test for continuous variables and Fisher’s exact test for categorical variables. Statistical significance was set at *p* < 0.05.

In addition to *p*-values, we calculated effect sizes to quantify the magnitude of the differences. For within-group comparisons (baseline vs. 10th session), Cohen’s d was computed using the mean and standard deviation of change scores. For between-group comparisons at post-treatment, Cohen’s d was calculated using the pooled standard deviation of both groups. Ninety-five percent confidence intervals (95% CI) for all effect sizes were estimated using the standard error of d for independent or paired designs, as appropriate.

Given the pilot exploratory nature of this study, analyses were not adjusted for multiplicity and all *p*-values and effect sizes should be interpreted as hypothesis-generating.

## 3. Results

### 3.1. Participant Flow and Baseline Characteristics

**Figure 2:**
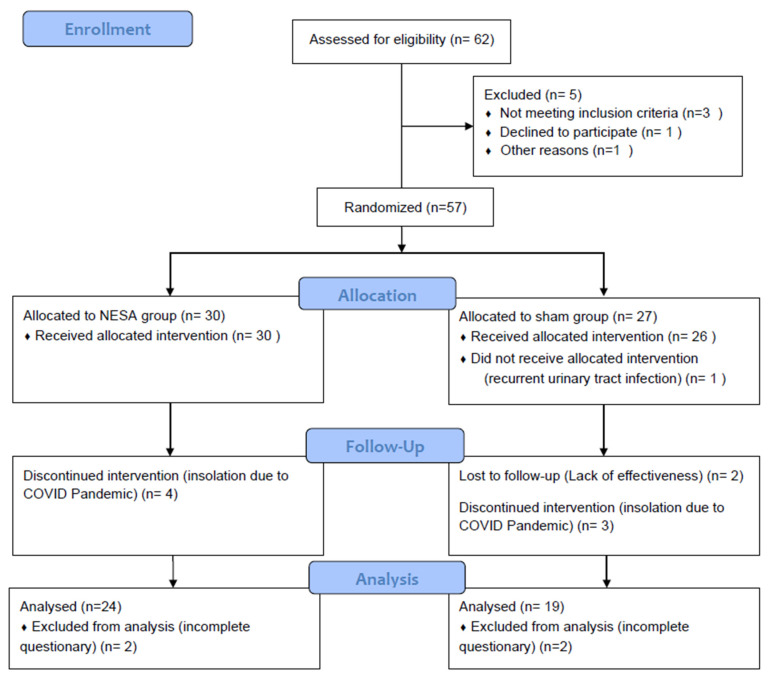
CONSORT flow diagram showing allocation to NESA and sham groups.

A total of 43 women were included in the per-protocol analysis, with 24 allocated to the NESA neuromodulation group and 19 to the sham group. Details of screening, randomization, and follow-up are presented in the CONSORT flow diagram (Figure 2).

The mean age of participants was 56.7 (12.5) years, ranging from 27 to 77 years. Overall, 41.3% (*n* = 19) were younger than 55 years. Baseline demographic and clinical characteristics, including age, body mass index, smoking status, and educational level, were comparable between the two groups, with no statistically significant differences (Table 1).

### 3.2. Bladder Diary Outcomes

#### 3.2.1. Within-Group Changes

In the NESA group, there were significant improvements over time in several bladder diary parameters (Table 2). The mean daily micturition (MDM) decreased from 9.19 (SD 3.32) at baseline to 8.07 (SD 2.74) after 10 sessions (*p* = 0.043). The number of incontinent episodes (NIE) also showed a significant reduction across study visits (*p* = 0.015). Mean voided volume (MVV) increased numerically over time, from 157.9 (SD 53.0) mL at baseline to 170.8 (SD 78.0) mL post-treatment, although this change was not statistically significant (*p* = 0.687). No significant change was observed in the number of nocturnal voids (NNV) (*p* = 0.448).

In the sham group, small fluctuations were observed in diary variables over time without consistent improvement. The pattern was compatible with a modest transient change followed by a rebound towards baseline values, particularly in daily micturition (Table 3, Supplementary Figure S1).

Effect size analysis showed small-to-moderate improvements across most outcomes in the NESA group. Specifically, mean daily micturitions demonstrated a small-to-moderate within-group effect (d = −0.42), while sleep-related measures exhibited moderate effect sizes (PSQI d = −0.52; ISI d = −0.44). Detailed effect size estimates are provided in Table 2 and Supplementary Table S1.

#### 3.2.2. Between-Group Comparison

At post-treatment, the only significant between-group urinary diary difference was observed for mean daily micturition (Table 3). By contrast, the remaining urinary diary variables did not show significant between-group differences at the end of treatment. Women receiving NESA showed a reduction in MDM from baseline to post-treatment, whereas those in the sham group demonstrated a slight increase (*p* = 0.043 for the between-group comparison).

The between-group standardized mean difference for post-treatment mean daily micturitions indicated a large effect size favoring NESA (d = −0.97; 95% CI −1.61 to −0.33), whereas effect sizes for other bladder diary variables (NIE, NNV, MVV) were small and their confidence intervals crossed zero. Sleep outcomes showed moderate effects favoring NESA (PSQI d = −0.39; ISI d = −0.48), consistent with the statistically significant between-group differences. Comprehensive effect size estimates are summarized in Supplementary Table S1.

### 3.3. Patient-Reported Urinary Outcomes

In the NESA group, scores on the ICIQ-UI SF (symptom severity) decreased significantly over time, from 11.21 (SD 5.87) at baseline to 8.08 (SD 6.24) at the end of treatment (*p* = 0.007). The associated quality-of-life score (ICIQ-QoL) showed a similar pattern, improving from 6.13 (SD 3.34) to 4.50 (SD 3.49) (*p* = 0.004) (Table 2).

B-SAQ Symptom and Bother scores also improved significantly within the NESA group (*p* = 0.009 and *p* = 0.038, respectively), indicating a reduction in urinary symptom burden and perceived bother (Table 2).

In the sham group, modest numerical changes were seen in ICIQ-UI SF and B-SAQ scores, but no consistent or statistically significant improvements over time were observed (Table 3).

However, when directly comparing NESA and sham groups, between-group differences in ICIQ-UI SF and B-SAQ outcomes did not reach statistical significance at the end of treatment (Table 3). Improvements in urinary symptom questionnaires therefore appeared more pronounced within the NESA group but should be interpreted as exploratory.

### 3.4. Sleep Outcomes

Sleep-related measures showed the most robust and consistent changes favoring NESA neuromodulation.

In the NESA group, the PSQI total score improved significantly from 7.90 (SD 4.94) at baseline to 5.40 (SD 3.46) post-treatment (*p* = 0.003), indicating better overall sleep quality (Table 2). The ISI score decreased from 10.43 (SD 7.17) to 7.48 (SD 7.08) (*p* = 0.002), reflecting reduced insomnia severity.

In the sham group, PSQI and ISI scores showed slight numerical improvements over time but without reaching statistical significance (Table 3).

Between-group comparisons demonstrated significant differences in favor of NESA for both PSQI and ISI at post-treatment, with *p* = 0.001 for each outcome (Table 3, Supplementary Figure S2). These findings suggest a consistent improvement in sleep quality and insomnia severity in women treated with NESA compared with sham stimulation.

### 3.5. Safety

No adverse events related to the intervention were reported in either group throughout the study period. All participants tolerated the sessions well, and no patient discontinued treatment due to discomfort or device-related complications.

## 4. Discussion

This pilot study presents the first investigation of non-invasive NESA neuromodulation in overactive bladder syndrome. In this pilot sample, the most consistent between-group signal favoring NESA was observed in sleep-related outcomes, together with a significant reduction in mean daily micturition. By contrast, other urinary diary variables and patient-reported urinary outcomes showed smaller, less consistent, or non-significant between-group differences. Therefore, any interpretation of urinary benefit beyond mean daily frequency should remain cautious. As with other randomized studies, the placebo effect played a role in the results. This phenomenon prompts consideration of the nervous system’s role in OAB pathophysiology and its impact on clinical trials. Additional research is required to determine the underlying mechanisms of the placebo effect in OAB [20]. As this was a pilot study, we plan to increase the follow-up time and number of participants in future research to mitigate this phenomenon. This issue is particularly relevant in OAB research, where placebo responses are often substantial and may influence both subjective symptom reporting and behavioral diary measures. In this context, the modest fluctuations observed in the sham group reinforce the need for cautious interpretation of small treatment effects in exploratory studies.

In line with the descriptive findings, effect size analysis reinforced that the only clinically meaningful bladder-related difference between groups was the reduction in mean daily micturitions, which showed a large, standardized effect (d = −0.97) in this pilot sample. All other urinary diary and questionnaire measures showed small and non-significant effects, indicating that potential improvements were modest and imprecise in this sample. By contrast, sleep-related outcomes demonstrated moderate standardized effects favoring NESA, suggesting that the intervention may exert more consistent influence on autonomic or sleep-regulatory mechanisms than on bladder-specific symptoms. These findings support the exploratory nature of this pilot trial and the need for larger studies with objective physiological endpoints.

Although the reduction in mean daily micturition reached statistical significance, its absolute magnitude was modest and should therefore be interpreted with caution from a clinical perspective. In this sense, the present findings are better understood as a preliminary signal of potential benefit rather than as evidence of a clearly established clinically meaningful effect. Future trials should define a priori thresholds of clinical relevance and compare the magnitude of benefit with established therapies used in OAB management.

In addition, the absence of multiplicity adjustment across multiple endpoints increases the possibility of type I error. For this reason, statistically significant findings should be interpreted as exploratory signals rather than as definitive evidence of efficacy.

Currently, there are several therapies available for the treatment of overactive bladder: in the field of electrotherapy, we have posterior tibial nerve stimulation, which can be performed superficially (PTNS) or percutaneously (EPTP), or sacral neuromodulation (SNM). From a more conservative point of view, lifestyle modifications, anticholinergic medication or pelvic floor exercises (PFE) are used. Several meta-analyses indicate that there is no statistically significant difference between superficial and percutaneous stimulation or anticholinergic drugs for the non-surgical treatment of people with OAB [21]. This treatment is safe, low-cost, and easy to apply and administer. New developments in neuromodulation include new devices based on the neurostimulation of the tibial nerve with permanent surgical implantation. Te Dorsthorst et al. (2020) [22] describe techniques and devices (implants surgery) published, describing the advantages and disadvantages of each of them. They analyze the little free devices that are easy to implement in a less invasive way, and the possibility to modify the used programs. NESA is a non-invasive neuromodulation technique that is easy to apply. Unlike other electrical stimulations, it offers the possibility of applying different treatment programs in the same session, allowing for global treatment of the patient. This can improve important aspects of quality of life, such as sleep quality.

It is known from experience in other fields that the use of NESA may influence the autonomic nervous system, improving aspects that are dependent on the autonomic system, such as cardiac variability or constipation. Autonomic nervous system involvement is known to play a role in the etiopathogenesis of overactive bladder syndrome. However, there is currently no marker to indicate the degree of involvement of the autonomic nervous system, the affected patients, or the extent to which it affects the functioning of the lower urinary system. This pilot study used a sample of women over 18 years of age. However, this may have spread the age of our sample too thinly, resulting in different types of etiopathogenesis in overactive bladder. In our next research work, we aim to define which patients might have a greater involvement of the autonomic nervous system, as they will likely benefit more from the NESA neuromodulation system.

The intervention group’s improvement in sleep deserves special mention. It is known that patients with overactive bladder syndrome experience a decrease in sleep quality, even without nocturia 4.5. A US survey of 11,000 patients [23] found that poor sleep quality and depression are among the consequences of overactive bladder syndrome. The sleep-related findings observed in this study support further investigation of NESA neuromodulation as a potential strategy for addressing sleep disturbances associated with OAB. Therefore, it is crucial to continue researching sleep medicine to improve the quality of life for these patients.

This pilot study yielded preliminary findings compatible with a possible role of autonomic-related mechanisms, particularly regarding sleep-related outcomes. However, further research with larger samples and objective physiological measures is needed to validate this hypothesis. In the future, it is necessary to conduct comparative studies between different types of electrostimulations, such as PTNS or SNM, in contrast to non-invasive neuromodulation NESA. Some limitations of the study are worth noting. For instance, the sample size was reduced due to losses suffered during the study, coinciding with the period of confinement in Spain caused by the pandemic. Moreover, beyond the significant between-group effect observed for mean daily micturitions, other diary variables such as nocturnal micturitions did not reach statistical significance despite potentially clinically relevant numerical trends. These findings emphasize the need for larger trials to more precisely characterize the impact of NESA neuromodulation on different aspects of bladder function.

An additional limitation is that the efficacy analysis was conducted on a per-protocol basis. Although understandable in an exploratory pilot context, the exclusion of participants after randomization may have introduced attrition bias and could have led to an overestimation of treatment effects.

The follow-up period was limited to the treatment phase, with no post-treatment assessment of durability. Since OAB is a chronic condition, the absence of longer follow-up limits conclusions regarding the persistence of any observed benefit.

Furthermore, the confidence intervals around most effect sizes were wide and crossed zero, reflecting the limited precision inherent to the sample size. These estimates should therefore be interpreted as preliminary, serving primarily to guide future sample size calculations rather than as definitive evidence of clinical efficacy.

The consistency of the sleep-related findings suggests that this domain may represent a particularly relevant target for future research on NESA neuromodulation in women with OAB, especially if combined with longer follow-up and objective physiological measures.

## 5. Conclusions

Non-invasive NESA neuromodulation was safe and showed preliminary signals of benefit in this pilot randomized trial, particularly for mean daily micturition and sleep-related outcomes. However, given the pilot design, the modest sample size, and the per-protocol analysis, these findings should be interpreted cautiously. Further randomized trials with larger samples, intention-to-treat analyses, and longer follow-up are required before drawing definitive conclusions regarding clinical efficacy.

## Figures and Tables

**Table 1:** Baseline demographic and clinical characteristics.

Variable	NESA Group (*n* = 24)	Sham Group (*n* = 19)	*p*-Value
Age, years (mean ± SD)	58.63 ± 10.69	53.33 ± 13.39	0.262
BMI, kg/m^2^ (mean ± SD)	27.28 ± 5.62	26.76 ± 7.32	0.093
Tobacco use, *n* (%)			
Smoker	1 (4%)	4 (19.0%)	0.614
Ex-smoker	3 (12%)	3 (14.3%)	0.715
Never smoked	20 (84%)	12 (66.7%)	0.121
Education level, *n* (%)			
Elementary	7 (29%)	2 (9.5%)	0.462
Secondary	14 (58.3%)	12 (57.1%)	0.887
University	3 (12.5%)	5 (33.3%)	0.293

**Table 2:** Changes over time in the NESA group.

Variable	Baseline Mean (SD)	5th Session	10th Session	*p* (time)	d Pre–Post	IC95%
NIE	2.46 (2.98)	1.49 (3.34)	1.94 (3.52)	0.015	−0.18	−0.57 to 0.20
NNV	1.21 (0.93)	1.24 (1.38)	1.11 (1.20)	0.448	−0.08	−0.46 to 0.30
MDM	9.19 (3.32)	9.29 (2.51)	8.07 (2.74)	0.043	−0.42	−0.83 to −0.02
MVV (mL)	157.9 (53.0)	163.4 (64.1)	170.8 (78.0)	0.687	+0.20	−0.22 to 0.62
ICIQ-UI SF	11.21 (5.87)	9.83 (6.49)	8.08 (6.24)	0.007	−0.48	−0.90 to −0.06
ICIQ-QoL	6.13 (3.34)	5.17 (3.37)	4.50 (3.49)	0.004	−0.51	−0.93 to −0.10
B-SAQ Symptom	6.57 (3.10)	5.17 (2.86)	5.13 (3.33)	0.009	−0.39	−0.81 to 0.02
B-SAQ Bother	7.50 (3.18)	6.50 (3.15)	6.38 (3.31)	0.038	−0.35	−0.77 to 0.07
PSQI	7.90 (4.94)	6.15 (4.16)	5.40 (3.46)	0.003	−0.52	−0.94 to −0.11
ISI	10.43 (7.17)	7.87 (6.42)	7.48 (7.08)	0.002	−0.44	−0.86 to −0.02

**Table 3:** Interventional group (NESA) and placebo comparison after 5th and 10th session.

Variable	NESA Baseline Mean (SD)	NESA 5th	NESA 10th	Placebo Baseline	Placebo 5th	Placebo 10th	*p* * (10th)	d (Post)	IC95% d
NIE	2.46 (2.98)	1.49 (3.34)	1.94 (3.52)	2.60 (3.65)	2.76 (3.51)	2.26 (3.21)	ns	−0.13	−0.60 to 0.34
NNV	1.21 (0.93)	1.24 (1.38)	1.11 (1.20)	1.18 (1.21)	1.77 (1.80)	1.52 (1.24)	ns	−0.22	−0.69 to 0.25
MDM	9.19 (3.32)	9.29 (2.51)	8.07 (2.74)	10.56 (2.95)	9.94 (2.82)	11.03 (3.42)	0.043	−0.97	−1.61 to −0.33
MVV (mL)	157.9 (53.0)	163.4 (64.1)	170.8 (78.0)	139.0 (44.4)	145.5 (53.0)	149.8 (76.1)	ns	+0.18	−0.29 to 0.65
ICIQ-UI SF	11.21 (5.87)	9.83 (6.49)	8.08 (6.24)	10.32 (6.14)	9.79 (6.03)	8.53 (6.51)	ns	−0.10	−0.57 to 0.37
ICIQ-QoL	6.13 (3.34)	5.17 (3.37)	4.50 (3.49)	5.58 (3.52)	5.47 (3.47)	4.32 (3.51)	ns	0.00	−0.47 to 0.47
B-SAQ Symptom	6.57 (3.10)	5.17 (2.86)	5.13 (3.33)	7.32 (2.36)	6.84 (3.22)	6.16 (3.25)	ns	−0.15	−0.62 to 0.32
B-SAQ Bother	7.50 (3.18)	6.50 (3.15)	6.38 (3.31)	8.47 (2.78)	7.89 (3.93)	6.63 (3.91)	ns	−0.09	−0.56 to 0.38
PSQI	7.90 (4.94)	6.15 (4.16)	5.40 (3.46)	7.46 (3.23)	7.15 (3.13)	6.62 (2.76)	0.001	−0.63	−1.20 to −0.05
ISI	10.43 (7.17)	7.87 (6.42)	7.48 (7.08)	12.37 (7.20)	10.84 (6.91)	10.89 (7.07)	0.001	−0.59	−1.16 to −0.02

* *p*-value refers to the between-group comparison at post-treatment (10th session).

## Data Availability

Data are available upon reasonable request from the corresponding author due to privacy, ethical, and legal restrictions associated with human subject questionnaire data.

## Supplementary Materials

The following supporting information can be downloaded at: https://www.mdpi.com/article/10.3390/medicina62050936/s1, Figure S1: Evolution of 24-h micturition diary outcomes; Figure S2: Changes in sleep quality outcomes; Table S1: Standardized mean differences between NESA and sham groups.

## Abbreviations

The following abbreviations are used in this manuscript:

OAB	Overactive Bladder
ICS	International Continence Society
UUI	Urinary Urgency Incontinence
UTI	Urinary Tract Infection
EPIC	Epidemiology of Incontinence
SNS	Sacral Nerve Stimulation
P-PTNS	Percutaneous Posterior Tibial Nerve Stimulation
TENS	Transcutaneous Electrical Nerve Stimulation
SARS	Sacral Anterior Root Stimulation
DGN	Dorsal Genital Nerve
EG	Experimental Group
PG	Placebo Group
DM3d	Three-Day Voiding Diary
UI	Urinary Incontinence
NIE	Number of Incontinent Episodes
NNV	Number of Nightly Voids
MDM	Mean Daily Micturition
MVV	Mean Volume Voided
B-SAQ	Bladder Overactive Control Self-Assessment Questionnaire
ICIQ_UI SF	International Consultation on Incontinence Questionnaire on Urinary Incontinence (Short Form)
PSQI	Pittsburgh Sleep Quality Index
ISI	Insomnia Severity Index
ICIQ PROMS	International Consultation on Incontinence Questionnaire Patient-Reported Outcome Measures
