# Supplementary material for: Noninvasive NESA Microcurrent Neuromodulation for Refractory Overactive Bladder in Women: A Triple-Blind, Randomized, Sham-Controlled Pilot Trial
Source: Medicina (Kaunas). 2026 May 11;62(5):936. doi: 10.3390/medicina62050936 (PMC13208972; doi:10.3390/medicina62050936)
Supplement: Supplementary file 1 [file medicina-62-00936-s001.zip › Supplementary Table S1.pdf]

Supplementary Table S1. Standardized mean differences (Cohen's d) between NESA and placebo groups at baseline and at the 10th session

| Variable | d baseline | 95% CI d (baseline) | d post (10th session) | 95% CI d (post) |
|---|---|---|---|---|
| **NIE** | –0.04 | –0.64 to 0.56 | –0.09 | –0.70 to 0.51 |
| **NNV** | +0.03 | –0.57 to 0.63 | –0.34 | –0.94 to 0.27 |
| **MDM** | –0.43 | –1.04 to 0.18 | **–0.97** | **–1.61 to –0.33** |
| **MVV (mL)** | +0.38 | –0.23 to 0.99 | +0.27 | –0.33 to 0.88 |
| **ICIQ-UI SF** | +0.15 | –0.45 to 0.75 | –0.07 | –0.67 to 0.53 |
| **ICIQ-QoL** | +0.16 | –0.44 to 0.76 | +0.05 | –0.55 to 0.65 |
| **B-SAQ Symptom** | –0.27 | –0.87 to 0.34 | –0.31 | –0.92 to 0.29 |
| **B-SAQ Bother** | –0.32 | –0.93 to 0.28 | –0.07 | –0.67 to 0.53 |
| **PSQI** | +0.10 | –0.50 to 0.71 | –0.39 | –0.99 to 0.22 |
| **ISI** | –0.27 | –0.88 to 0.34 | –0.48 | –1.09 to 0.13 |

Note: Cohen's d calculated using pooled SD for independent groups; negative values favour NESA (lower symptom scores)
