# Supplementary figures and images for: Noninvasive NESA Microcurrent Neuromodulation for Refractory Overactive Bladder in Women: A Triple-Blind, Randomized, Sham-Controlled Pilot Trial
Source: Medicina (Kaunas). 2026 May 11;62(5):936. doi: 10.3390/medicina62050936 (PMC13208972; doi:10.3390/medicina62050936)

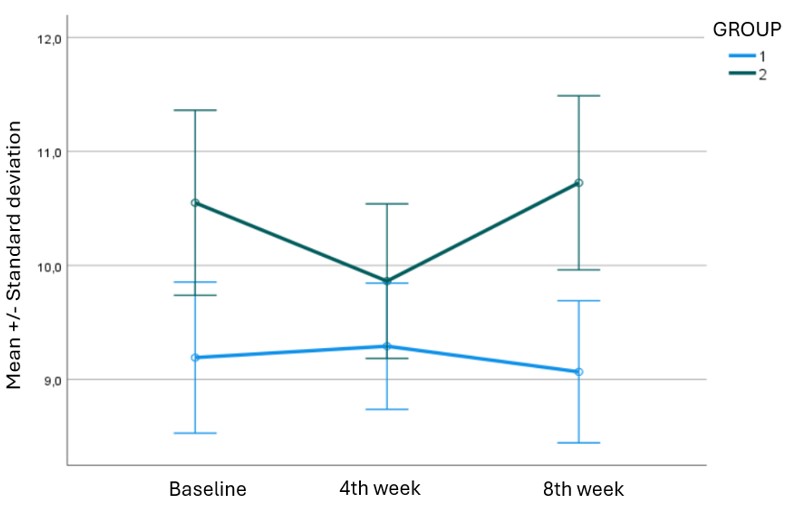

Supplement: Supplementary file 1 [file medicina-62-00936-s001.zip › Supplementary Figure S1.jpg]

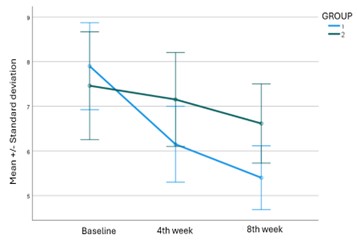

Supplement: Supplementary file 1 [file medicina-62-00936-s001.zip › Supplementary Figure S2.jpg]
